# VHLdb: A database of von Hippel-Lindau protein interactors and mutations

**DOI:** 10.1038/srep31128

**Published:** 2016-08-11

**Authors:** Francesco Tabaro, Giovanni Minervini, Faiza Sundus, Federica Quaglia, Emanuela Leonardi, Damiano Piovesan, Silvio C. E. Tosatto

**Affiliations:** 1 Department of Biomedical Sciences and CRIBI Biotechnology Center, University of Padova, Viale G. Colombo 3, 35121, Padova, Italy; 2 Institute of Biosciences and Medical Technology, Tampere, Finland; 3 Department of Woman and Child Health, University of Padua, 35131 Padua, Italy; 4 CNR Institute of Neuroscience, Padova, Italy

## Abstract

Mutations in von Hippel-Lindau tumor suppressor protein (pVHL) predispose to develop tumors affecting specific target organs, such as the retina, epididymis, adrenal glands, pancreas and kidneys. Currently, more than 400 pVHL interacting proteins are either described in the literature or predicted in public databases. This data is scattered among several different sources, slowing down the comprehension of pVHL’s biological role. Here we present VHLdb, a novel database collecting available interaction and mutation data on pVHL to provide novel integrated annotations. In VHLdb, pVHL interactors are organized according to two annotation levels, manual and automatic. Mutation data are easily accessible and a novel visualization tool has been implemented. A user-friendly feedback function to improve database content through community-driven curation is also provided. VHLdb presently contains 478 interactors, of which 117 have been manually curated, and 1,074 mutations. This makes it the largest available database for pVHL-related information. VHLdb is available from URL: http://vhldb.bio.unipd.it/.

Von Hippel-Lindau (VHL) syndrome is a hereditary predisposition to develop several cancers resulting from pathological inactivation of the von Hippel-Lindau protein (pVHL)^1^,^2^,^3^. pVHL is the product of the same gene located on chromosome 3p25 and constantly transcribed in both fetal and adult tissues^4^. Two different alternatively-spliced isoforms were initially identified^5^. pVHL30 contains all 213 residues of the VHL gene, whereas pVHL19 lacks the first 53 residues due to an alternative translation start site^5^. Both isoforms are biologically active, binding elongins B and C and cullin 2 to form an ubiquitin E3 ligase complex known as VCB^6^,^7^. The main pVHL function is ubiquitin-mediated degradation of hypoxia-inducible factor 1-alpha (HIF-1α)^3^ and pVHL activity is crucial in the oxygen sensing pathway. Under physiological oxygen concentrations, pVHL targets HIF-1α for proteosomal degradation. In hypoxic conditions HIF-1α escapes ubiquitin-mediated proteolysis and translocates to the nucleus, where it activates many genes involved in angiogenesis, oxidative metabolism, cell survival, and cancer progression^3^,^8^. Several other cellular functions not directly related to the pVHL/HIF-1α axis are also reported^9^,^10^,^11^,^12^,^13^,^14^,^15^, e.g. external matrix deposition, drawing a complex scenario for pVHL in cells and tissues. Numerous efforts addressed the specific pVHL molecular pathway^16^,^17^, describing pVHL as a molecular hub, mediating interactions with more than 200 different proteins^18^. Recently, a third pVHL isoform of unknown function was reported in the literature^19^, making an interpretation of pVHL’s molecular role even harder. pVHL has no significant sequence identity to other human proteins, but is well conserved within mammals^20^. Even between mammals pVHL shows important differences. The main distinction resides in the N-terminus of pVHL30, which is disordered^21^ and contains many copies of an acidic pentamer in human and other higher primates, while being shorter and lacking the disordered N-terminal tail region in lower mammals^22^. VHL syndrome is characterized by the development of several generally benign tumors, which affect specific target organs, such as the retina, epididymis, adrenal glands, pancreas and kidneys^1^,^23^,^24^. It is considered a severe autosomal dominant genetic condition with inheritance of one in over 35,000^25^. Defects of pVHL function are not limited to the sole VHL syndrome. It is thought that pVHL tumor suppressor loss of function is present in ca. 75% of clear cell renal cellular carcinomas (ccRCC) not directly related to VHL syndrome^26^. Recent studies also suggest a role for pVHL in p53 tumor suppressor regulation^27^,^28^. Kidney-specific pVHL inactivation causes the development of kidney cysts in a mouse model^29^, while reintroduction of a wild type gene interrupts malignant progression^30^. A number of experimental and *in silico* data of proteins involved in pVHL tumorigenesis is reported^9^,^13^ and contained in large databases, such as IntAct^18^, STRING^31^ and BioGRID^32^. It is thought that pVHL has at least four different protein-protein interaction interfaces (A to D)^13^. Several specific interactors were found for each interface and correlation with functions other than oxygen sensing, such as DNA-damage repair^33^, microtubule dynamics^34^ and oxidative metabolism, reinforce the pivotal role of pVHL. As the amount of details known about pVHL function is rapidly increasing, the multiple pVHL roles may confound our understanding of this complex protein. Knowledge is usually derived from freely accessible protein sequence and function databases. Although valuable, these universal resources are generalist by design, yielding a strong fragmentation of the huge amount of pVHL data. For a non-bioinformatician, scattered information represents one of the biggest hurdles, slowing down a holistic understanding of the pVHL biological role. Here we present VHLdb, a novel resource providing expert curation for the pVHL tumor suppressor. The database was primarily designed to be effective for a non-expert, making information retrieval easier. Overall, VHLdb accounts for 478 unique interactors in two curation levels (manual and automatic), with data retrieved from different sources. Detailed information on the pVHL interaction interface and post-translational modifications were also included. A feedback function allows inclusion of novel information from experts in the field wishing to contribute annotation on interactors or mutations. Finally, a downloading tool is also provided for data sharing.

## Database Description

### Mutation data

**Figure 1:**
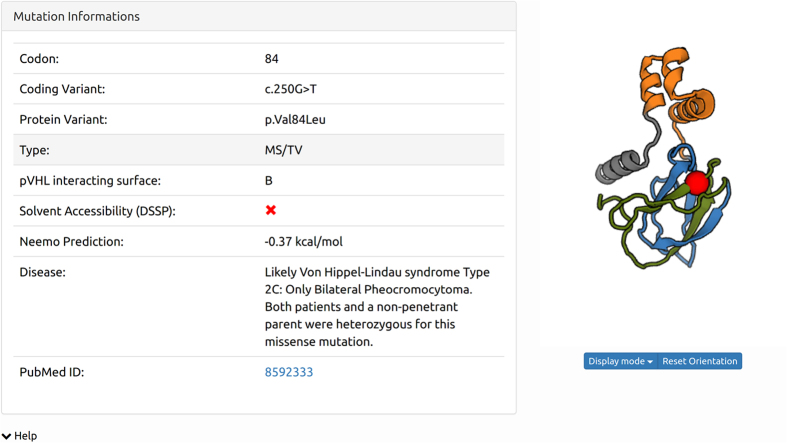
Example of a mutation as displayed in VHLdb. For each mutation all available details are listed (i.e. coding variant, effect on protein, type of mutation, pVHL surface involved, solvent accessibility, phenotype, thermodynamic predictions and reference) and visualized as a red sphere on the surface-colored pVHL structure.

Germline and somatic mutations have been collected from^35^,^36^,^37^,^38^, integrated^39^,^40^,^41^ and annotated with predictions on protein stability. The final dataset is made up of 1,074 mutations and, to the best of our knowledge, represents the largest publicly available repository of pathogenic pVHL variants. An example of mutation details is given in Table 1 and Fig. 1. Where possible, a pVHL interacting surface has been defined for each mutation. E.g. frameshift mutations cannot be assigned to any surface due to their intrinsic nature. Solvent accessibility has been computed for each mutation using DSSP^42^ and mutated residues are defined exposed when at least 20% of their surface is accessible to solvent. Bluues^43^ and NeEMO^44^ have been run on all possible mutations, using the pVHL 3D structure with PDB code 1LM8 as reference. Current pVHL 3D structures cover only the structured part of the protein (i.e. alpha- and beta-domains), lacking the first 60 residues which form an intrinsically disordered tail. Pathogenicity assessment for mutations in this segment was not included in VHLdb to avoid the risk of erroneous interpretation from low confidence predictions. Bluues^43^ calculates the electrostatic properties of a protein and is able to predict electrostatic properties of mutated solvent exposed residues. NeEMO^44^ evaluates stability changes caused by amino acid substitutions using a machine learning based approach from structure. It has been run on all point mutations of the crystallized protein, i.e. again excluding only the N-terminus.

### pVHL interactome

**Figure 2:**
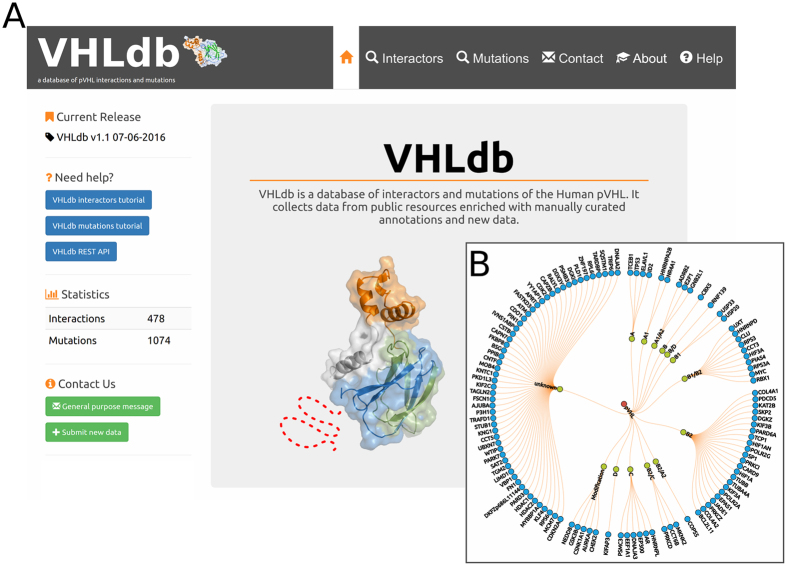
VHLdb home page and VHLdb manually curated interactors set. (**A**) VHLdb home page. On the left, a column with version, statistics and useful links. On the right, a clickable image which redirects to the pVHL interacting proteins page. (**B**) Manually curated pVHL interacting proteins sorted by interacting surface. Proteins labelled with modification are the ones which bind pVHL upon post-translational modification. Proteins for which no interacting surface could be determined are labeled unknown.

The pVHL interactome has been defined starting from searches in publicly available databases. VHLdb contains two levels of annotation for interactors, automatic and manual (Fig. 2). Automatic annotations are denoted by an empty silver star and build the overall pVHL interactome, albeit at a lower confidence level. Manually curated pVHL interactors, represented with a a gold star, have been annotated with the exact molecular details and their functional meaning.

The automatic pVHL interaction network has been generated with queries to the STRING^31^, BioGrid^45^, iHOP^46^, MIPS^47^ and IMEx^48^ databases. STRING and Biogrid are two of the most popular protein-protein interaction databases. The IMEx Consortium is a long-term coordination project which currently contains twelve interaction databases. MIPS is a database of mammalian interacting proteins while iHOPS is a text-mining based resource parsing the PubMed database for possible statements on a target protein interaction. Both are presented in a human readable format and their data is not associated with a confidence score. All interactions from IMEx, STRING are annotated with this measurement, while BioGrid interactions are poorly annotated. When available, this score is reported in the interactor page so the user can easily assess the interaction quality. The five resources have been queried through the standard user interface using the most general terms, i.e. “VHL” or “pVHL”. In all cases, only human interaction data was considered. The results from the different sources have been merged and processed to remove duplicates. Annotation from UniProt^49^, PDB^50^, Gene Ontology^51^, Pfam^52^ and MobiDB^21^ has been added. Searches in interaction databases allowed us to build the full network, currently containing 478 proteins.

### Manually curated pVHL interactions

**Figure 3:**
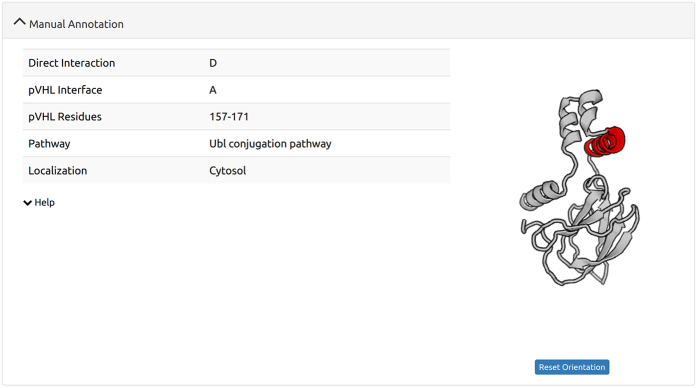
Example of manually curated interaction annotation. For each of the manually curated pVHL interacting proteins, informations from the manual curation process are listed and, if the pVHL interacting residues are known, displayed in an interactive window.

The manually curated high quality pVHL interaction network is currently composed of 117 proteins. 35 come from a previous publication^13^ while the others have been annotated and are presented in this work (see Table 2). Data curation was performed by each expert following an in-house standardized protocol to guarantee reproducibility and correctness. In detail, the manual curation workflow considers a preliminary search in Pubmed^53^ and Uniprot^49^ using pVHL-related keywords (e.g. “VHL syndrome”, “pVHL AND ccRCC”) adapted to the interactor under investigation. Keywords were manually selected by curators using the most common keywords found in the VHL syndrome literature, e.g. angiogenesis, proteasome degradation, oxygen sensing. In case of proteins with different synonymous names (e.g. the EGLN protein family also known as PHD) multiple searches were performed. The final nomenclature for each VHLdb entry was chosen using the official HGNC consortium name. Interaction details have been manually extracted from the literature. Pubmed has been searched for papers describing either structural details of the interaction (e.g. pVHL and target protein residues, sequence motifs and domains) and their functional implications. An example of structural details of the interaction is given in Fig. 3. Upon identification, each interactor has been analyzed with Consurf 54 to assess sequence conservation as well as PRISM^55^ and Crescendo^56^ to predict the spatial localization of the interaction at the residue level. Presence of linear sequence motifs, known to be relevant in protein-protein interactions, post-translational modification or enzymatic cleavage was performed with ELM^57^. The interaction surface was assigned following our classification^13^ as summarized in Table 3.

### Implementation

**Figure 4:**
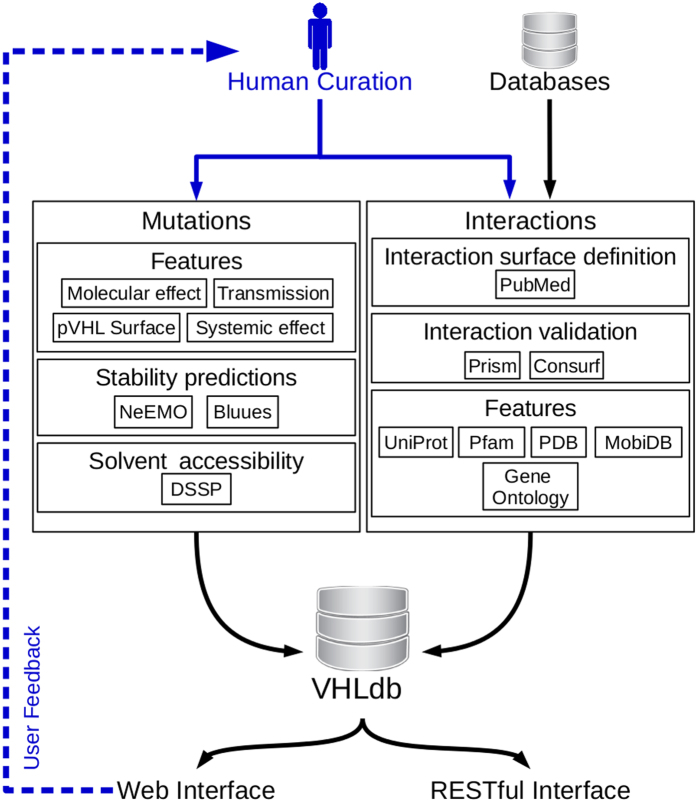
Schematic representation of the VHLdb implementation schema. Black arrows represent the data flow from curators to end users. The gray arrow represents the feedback function VHLdb offers to the end users to report an entry, submit new data o more simply contact the curators.

VHLdb uses separate modules for data management, processing and presentation. Figure 4 shows a schematic representation of the whole application. To eliminate the need for data conversion, simplifying development and maintenance, all modules share the JSON (JavaScript Object Notation) format to exchange data. The MongoDB database engine is used for storage and Node.js as middleware between data and presentation. VHLdb exposes its resources through a RESTful interface, using the Restify library for Node.js. At the time of writing, VHLdb supports a custom REST API, the search-route, as detailed in the Help page. The user interface is implemented using the Angular.js framework and Bootstrap library. These libraries provide a mobile-ready interface, allowing VHLdb to be natively accessed from any kind of device. Structural annotations are displayed with the Web-GL based molecular viewer PV^58^. Custom molecular views have been developed. An “interaction viewer” has been implemented in the entry page to display interaction data and a “mutation viewer” has been implemented in the mutations page. The former allows the user to visualize the pVHL residues interacting with a manually curated interacting protein by highlighting the interacting region on the pVHL structure. The latter displays the location of any mutation on the pVHL structure as a sphere, allowing the user to visually access the structural location of a mutation. VHLdb allows direct download of all pVHL interactions, as well as mutations. The database offers both a graphical web interface and RESTful web services from the URL: http://VHLdb.bio.unipd.it/.

## Results

### Using VHLdb

VHLdb offers simple yet powerful ways to access its data. First, the navigation bar on top of the home page allows the user to access the mutation or interaction page. The home page features a clickable map, redirecting the user to interface-specific pVHL interaction lists (Fig. 2). The mutation page lists all coding variants (sorted by codon) in a user-friendly searchable, filterable and downloadable table, as well as the previously described mutation viewer. The interaction page features a graphical representation of the manually curated pVHL interaction network organized by interacting surface and a sortable, searchable and filterable table, similar to the mutations one listing all protein-protein interactions. The third element of this page is a table showing Gene Ontology (GO) enrichment analysis results for each surface and GO tree. This page allows download of the complete pVHL interaction set in four different formats (JSON, XML, CSV and TAB separated). Details of any protein can be accessed from the interaction page. This page shows all available annotations for a particular pVHL interacting protein including general annotations from UniProt, manually curated interaction details (if available), sequence annotation from Pfam and MobiDB, structure annotations from PDB, functional annotation from GO and references from PubMed. All these data can be downloaded in a protein-specific way in the formats specified above. A feeedback form is accessible from this page and can be used to report inconsistencies or suggest annotations for a specific pVHL interacting protein. Another way to give feedback and request data submission is the contact page accessible from the navigation bar, featuring two distinct submission forms, for general feedback and specific data submission requests. These messages are manually reviewed by our curators and after validation, i.e. confirmation of user-suggested literature, the proposed data will be added to VHLdb.

### VHLdb statistics

VHLdb collects data on 478 pVHL interacting proteins and 1,074 pathogenic somatic or germline pVHL mutations. In total, 117 of 478 pVHL interacting proteins were manually reviewed and constitute the core curated pVHL interaction network. The remaining proteins constitute the automated low confidence pVHL interaction network. For 62 proteins of the core set it was possible to identify the interacting surface (see Table 3). For 55 proteins it was possible to identify the pVHL residues involved in the interaction, and for 10 the residues of the interaction partner as well. For 51 proteins we also defined whether the interaction between pVHL and any other protein is direct or not. Table 2 shows a more detailed listing of the manually curated VHLdb protein set. Statistical analysis shows that the interactor distribution differs among the four pVHL interfaces. Interface A presents 9 exclusive interactors, distributed between sub-interfaces A1 and A2, and is known to bind elongins B and C and cullin 2 to form the VCB complex^6^. Interacting proteins in this region compete with elongins B and C, highlighting pVHL functions beyond the well known HIF-1α degradation. We also found that 190 mutations affect this area, yielding three different VHL phenotypes. E.g. Guanine nucleotide-binding protein subunit beta-2-like 1 and E2F transcription factor 1 (UniProt codes: P63244 and Q01094, respectively) are both known to promote cell cycle progression under different stimuli. A simple database search shows that the two proteins rely on the same interaction interface, suggesting a correlated role, at least for pVHL binding. Their interaction with the same pVHL surface suggests a pivotal pVHL role in controlling cell cycle progression under different stimuli and oxygen concentrations. Similar results were found for the remaining interaction interfaces. In detail, 39 interactors were found for interface B, 6 for interface C and one interactor for interface D, for a total of 827 different mutations distributed among interaction interfaces. Interface B is the HIF-1α binding region and characterized by the largest number of interactors. As a further example, we found that proteins such as tubulin beta, collagen alpha-1(IV) and kinesin bind sub-interface B2 showing that molecular details of functions related to endothelial matrix regulation^15^ should correspond to this specific interaction area.

## Conclusions

We have presented VHLdb, a novel database collecting curated information on pVHL interactors and mutation effects. It provides comprehensive information of pVHL interactors derived from different sources as a unique structured resource. As detailed information about VHL disease is rapidly increasing, this huge amount of information is scattered in different generalist resources and not promptly reachable by a non-expert user. We expect the VHLdb to be useful for both experimentalists seeking to study pVHL biology in greater details and clinicians aiming to understand the effects of novel pVHL variants. An intuitive pVHL oriented user interface was designed and four different output formats are provided to facilitate data retrieval. VHLdb is also effective for the qualitative study of pVHL pathogenic mutations and interacting proteins. From a total of 478 different interactors, 62 were mapped on the corresponding interaction interface. Moreover, 1,074 somatic and germline pathogenic mutations are reported, increasing the previous set of pathogenic pVHL mutations^35^. This can be particularly helpful for future mutation-correlation studies. Information in VHLdb may serve the scientific community to decipher data derived from tumor genome sequencing projects^59^ as well as to provide high quality data to be included in predictive genomics studies^60^. Updates such as error reports and submissions of new data to VHLdb are highly encouraged from the community through the implemented feedback function. For the future, it is envisaged the VHLdb will include more annotations, such as distinct causal relationships between mutations and affected pathways.

## Additional Information

**How to cite this article**: Tabaro, F. *et al.* VHLdb: A database of von Hippel-Lindau protein interactors and mutations. *Sci. Rep.* **6**, 31128; doi: 10.1038/srep31128 (2016).

## Figures and Tables

**Table 1:** Example of mutation data contained in VHLdb. For each mutation, codon, effect on protein, NeEMO prediction, disease and Pubmed id is given.

Codon	Effect	Surface	NeEMO (Kcal/mol)	Disease	PubMed ID	Curator
12	p.Glu12Asp	D		HB	21463266	E. L.
59	p.Pro59Ser	D		PH	21463266	E. L.
66	p.Val66Gly	B	0.17	CNS HB, PC, PH	21463266	E. L.
103	p.Pro103Ala	B	0.8	PH	21463266	E. L.
138	p.Pro138Thr	C	1.32	RHB, PH	21463266	E. L.
155	p.Val155Gly	A	0.19	RHB, RCC	21463266	E. L.
167	p.Arg167Leu	A	0.02	CNS HB, RHB, PH	21463266	E. L.

Curator name is reported in the last column. Abbreviations for the disease column: HB, hemangioblastoma, PH, pheochromocytoma, CNS HB, central nervous system hemangioblastoma, PC, prostate cancer, PH, pheochromocytoma, RHB, retinal hemangioblastoma, RCC, renal cell cancer. Abbreviations for the curator column: E. L., E. Leonardi.

**Table 2:** Example of manually curated interactors.

Uniprot ID	Gene Name	pVHL Surface	pVHL interacting residues	Interacting protein residues or domain	Associated disease	Curator
P45973	CBX5	B	PXVXL motif, β-domain		RCC, PH, HB	F. S., G. M.
P42771	CDKN2A		N-terminal Domain			F. S., G. M.
Q6N084	DKFZp686L11144				RCC	F. S., G. M.
P02751	FN1					E. L.
P49841	GSK3B	Modification	Ser68			E. L.
Q13547	HDAC1				RCC	F. S., G. M.
Q92769	HDAC2		Disordered			F. S., G. M.
P14866	HNRNPL	C				F. S., G. M.
Q02363	ID2	A	154–174			F.Q., G.M.
Q92845	KIFAP3	D	1–54			E. L.
O43474	KLF4			61–108	CCC	F. S., G. M.
P33993	MCM7					F. S., G. M.
Q9BQG0	MYBBP1A			Pro693		F. S., G. M.
Q8TEW0	PARD3				RCC	F. S., G. M.
Q8N2W9	PIAS4	B1/B2	54–120, β-domain	Carboxyl 46 amino acids	RCC	F. S., G. M.
A2A3R6	RPS6					F. S., G. M.
P61758	VBP1		87–213			E. L.
O14709	ZNF197		55–214	KRAB-A domain		F. S., G. M.

For each pVHL-interacting protein, Uniprot ID, Hugo name, interaction details, disease associated with interaction disruption, curators and their affiliation are reported. List of abbreviations for the disease column: CC, colorectal cancer, RCC, renal cell carcinoma. List of abbreviations for the curators column: F.S., F. Sundus, F.Q., F. Quaglia, G.M., G. Minervini, E. L., E. Leonardi.

**Table 3:** Distribution of VHLdb interactors and mutations by pVHL interacting surface.

Surface	Start	End	Interactors	Mutations
A	T154	D189	9	190
B	P60	R109	41	254
C	P104	I153	6	284
D	M1	R59	1	99
Unknown			55	280
Upon modification			5	
Total			117	1074

For each surface, start and end residues as well as the number of interactors and mutations are reported. The “upon modification’’ row indicates the number of proteins which bind the pVHL protein after it has been phoshorylated in some residue.

## References

[b1] Gnarra J. R. *et al.* Mutations of the VHL tumour suppressor gene in renal carcinoma. Nat. Genet. 7, 85–90 (1994). 7915601 10.1038/ng0594-85

[b2] Gossage L., Eisen T. & Maher E. R. VHL, the story of a tumour suppressor gene. Nat Rev Cancer 15, 55–64 (2015). 25533676 10.1038/nrc3844

[b3] Hon W.-C. *et al.* Structural basis for the recognition of hydroxyproline in HIF-1 alpha by pVHL. Nature 417, 975–978 (2002). 12050673 10.1038/nature00767

[b4] Stolle C. *et al.* Improved detection of germline mutations in the von Hippel-Lindau disease tumor suppressor gene. Hum. Mutat. 12, 417–423 (1998). 9829911 10.1002/(SICI)1098-1004(1998)12:6<417::AID-HUMU8>3.0.CO;2-K

[b5] Richards F. M., Schofield P. N., Fleming S. & Maher E. R. Expression of the von Hippel-Lindau disease tumour suppressor gene during human embryogenesis. Hum. Mol. Genet. 5, 639–644 (1996). 8733131 10.1093/hmg/5.5.639

[b6] Kibel A., Iliopoulos O., DeCaprio J. A. & Kaelin W. G. Binding of the von Hippel-Lindau tumor suppressor protein to Elongin B and C. Science 269, 1444–1446 (1995). 7660130 10.1126/science.7660130

[b7] Iwai K. *et al.* Identification of the von Hippel-lindau tumor-suppressor protein as part of an active E3 ubiquitin ligase complex. Proc. Natl. Acad. Sci. USA 96, 12436–12441 (1999). 10535940 10.1073/pnas.96.22.12436 PMC22941

[b8] Ivan M. *et al.* HIFalpha targeted for VHL-mediated destruction by proline hydroxylation: implications for O2 sensing. Science 292, 464–468 (2001). 11292862 10.1126/science.1059817

[b9] Frew I. J. & Krek W. pVHL: a multipurpose adaptor protein. Sci Signal 1, pe30 (2008). 18560019 10.1126/scisignal.124pe30

[b10] Gamper A. M. *et al.* Regulation of KLF4 Turnover Reveals an Unexpected Tissue-Specific Role of pVHL in Tumorigenesis. Molecular Cell 45, 233–243 (2012). 22284679 10.1016/j.molcel.2011.11.031 PMC3982234

[b11] Du J. *et al.* pVHL Negatively Regulates Antiviral Signaling by Targeting MAVS for Proteasomal Degradation. J Immunol 1500588 doi: 10.4049/jimmunol.1500588 (2015). 26179906

[b12] Xue J. *et al.* pVHL Mediates K63-Linked Ubiquitination of nCLU. PLoS ONE 7, e35848 (2012). 22532874 10.1371/journal.pone.0035848 PMC3332038

[b13] Leonardi E., Murgia a & Tosatto S. C. E. Adding structural information to the von Hippel-Lindau (VHL) tumor suppressor interaction network. FEBS letters 583, 3704–3710 (2009). 19878677 10.1016/j.febslet.2009.10.070

[b14] Ohh M. *et al.* The von Hippel-Lindau tumor suppressor protein is required for proper assembly of an extracellular fibronectin matrix. Mol. Cell 1, 959–968 (1998). 9651579 10.1016/s1097-2765(00)80096-9

[b15] Tang N., Mack F. & Haase V. pVHL function is essential for endothelial extracellular matrix deposition. Molecular and cellular \ldots 26, (2006). 10.1128/MCB.26.7.2519-2530.2006 PMC1430327 16537898

[b16] Lai Y., Song M., Hakala K., Weintraub S. T. & Shiio Y. Proteomic dissection of the von Hippel-Lindau (VHL) interactome. J. Proteome Res. 10, 5175–5182 (2011). 21942715 10.1021/pr200642c PMC3208728

[b17] Minervini G. *et al.* Design and analysis of a Petri net model of the Von Hippel-Lindau (VHL) tumor suppressor interaction network. PLoS ONE 9, e96986 (2014). 24886840 10.1371/journal.pone.0096986 PMC4041725

[b18] Kerrien S. *et al.* The IntAct molecular interaction database in 2012. Nucleic Acids Res. 40, D841–D846 (2012). 22121220 10.1093/nar/gkr1088 PMC3245075

[b19] Chesnel F. *et al.* The von Hippel–Lindau tumour suppressor gene: uncovering the expression of the pVHL172 isoform. Br J Cancer doi: 10.1038/bjc.2015.189 (2015). PMC4506380 26035699

[b20] Woodward E. R. *et al.* Comparative sequence analysis of the VHL tumor suppressor gene. Genomics 65, 253–265 (2000). 10857749 10.1006/geno.2000.6144

[b21] Potenza E., Di Domenico T., Walsh I. & Tosatto S. C. E. MobiDB 2.0: an improved database of intrinsically disordered and mobile proteins. Nucleic Acids Res. 43, D315–D320 (2015). 25361972 10.1093/nar/gku982 PMC4384034

[b22] Minervini G. *et al.* Isoform-specific interactions of the von Hippel-Lindau tumor suppressor protein. Sci Rep 5, 12605 (2015). 26211615 10.1038/srep12605 PMC4515828

[b23] Latif F. *et al.* Identification of the von Hippel-Lindau disease tumor suppressor gene. Science 260, 1317–1320 (1993). 8493574 10.1126/science.8493574

[b24] Shen H.-C. J. *et al.* Deciphering von Hippel-Lindau (VHL/Vhl)-associated pancreatic manifestations by inactivating Vhl in specific pancreatic cell populations. PLoS ONE 4, e4897 (2009). 19340311 10.1371/journal.pone.0004897 PMC2660574

[b25] Kim W. Y. & Kaelin W. G. Role of VHL gene mutation in human cancer. J. Clin. Oncol. 22, 4991–5004 (2004). 15611513 10.1200/JCO.2004.05.061

[b26] Young A. C. *et al.* Analysis of VHL Gene Alterations and their Relationship to Clinical Parameters in Sporadic Conventional Renal Cell Carcinoma. Clin. Cancer Res. 15, 7582–7592 (2009). 19996202 10.1158/1078-0432.CCR-09-2131 PMC2795746

[b27] Fels D. R. & Koumenis C. HIF-1alpha and p53: the ODD couple? Trends Biochem. Sci. 30, 426–429 (2005). 15996866 10.1016/j.tibs.2005.06.009

[b28] Jung Y.-S. *et al.* Loss of VHL promotes progerin expression, leading to impaired p14/ARF function and suppression of p53 activity. Cell Cycle 12, 2277–2290 (2013). 24067370 10.4161/cc.25371 PMC3755078

[b29] Rankin E. B., Tomaszewski J. E. & Haase V. H. Renal cyst development in mice with conditional inactivation of the von Hippel-Lindau tumor suppressor. Cancer Res. 66, 2576–2583 (2006). 16510575 10.1158/0008-5472.CAN-05-3241 PMC3514875

[b30] Iliopoulos O., Kibel A., Gray S. & Kaelin W. G. Tumour suppression by the human von Hippel-Lindau gene product. Nat Med 1, 822–826 (1995). 7585187 10.1038/nm0895-822

[b31] Franceschini A. *et al.* STRING v9.1: protein-protein interaction networks, with increased coverage and integration. Nucleic Acids Res. 41, D808–D815 (2013). 23203871 10.1093/nar/gks1094 PMC3531103

[b32] Stark C. *et al.* BioGRID: a general repository for interaction datasets. Nucl. Acids Res. 34, D535–D539 (2006). 16381927 10.1093/nar/gkj109 PMC1347471

[b33] Roe J.-S., Kim H.-R., Hwang I.-Y., Cho E.-J. & Youn H.-D. von Hippel-Lindau protein promotes Skp2 destabilization on DNA damage. Oncogene 30, 3127–3138 (2011). 21358672 10.1038/onc.2011.40

[b34] Lolkema M. P. *et al.* The von Hippel-Lindau tumor suppressor protein influences microtubule dynamics at the cell periphery. Exp. Cell Res. 301, 139–146 (2004). 15530850 10.1016/j.yexcr.2004.07.016

[b35] Nordstrom-O’Brien M. *et al.* Genetic analysis of von Hippel-Lindau disease. Hum. Mutat. 31, 521–537 (2010). 20151405 10.1002/humu.21219

[b36] Leonardi E., Martella M., Tosatto S. C. E. & Murgia A. Identification and in silico analysis of novel von Hippel-Lindau (VHL) gene variants from a large population. Ann. Hum. Genet. 75, 483–496 (2011). 21463266 10.1111/j.1469-1809.2011.00647.x

[b37] Rechsteiner M. P. *et al.* VHL gene mutations and their effects on hypoxia inducible factor HIFα: identification of potential driver and passenger mutations. Cancer Res. 71, 5500–5511 (2011). 21715564 10.1158/0008-5472.CAN-11-0757

[b38] Forbes S. A. *et al.* COSMIC: exploring the world’s knowledge of somatic mutations in human cancer. Nucleic Acids Res. 43, D805–D811 (2015). 25355519 10.1093/nar/gku1075 PMC4383913

[b39] Zhang B. *et al.* VHL gene mutation analysis of a Chinese family with non- syndromic pheochromocytomas and patients with apparently sporadic pheochromocytoma. Asian Pac. J. Cancer Prev. 16, 1977–1980 (2015). 25773797 10.7314/apjcp.2015.16.5.1977

[b40] Arunachal G. *et al.* Molecular Characterization of a Novel Germline VHL Mutation by Extensive In Silico Analysis in an Indian Family with Von Hippel-Lindau Disease. Genet Res Int 2016, 9872594 (2016). 27069690 10.1155/2016/9872594 PMC4812357

[b41] Sorrell A. D. *et al.* Clinical and functional properties of novel VHL mutation (X214L) consistent with Type 2A phenotype and low risk of renal cell carcinoma. Clin. Genet. 79, 539–545 (2011). 20560986 10.1111/j.1399-0004.2010.01464.x PMC2958253

[b42] Kabsch W. & Sander C. Dictionary of protein secondary structure: Pattern recognition of hydrogen-bonded and geometrical features. Biopolymers 22, 2577–2637 (1983). 6667333 10.1002/bip.360221211

[b43] Walsh I. *et al.* Bluues server: electrostatic properties of wild-type and mutated protein structures. Bioinformatics 28, 2189–2190 (2012). 22711791 10.1093/bioinformatics/bts343

[b44] Giollo M., Martin A. J., Walsh I., Ferrari C. & Tosatto S. C. NeEMO: a method using residue interaction networks to improve prediction of protein stability upon mutation. BMC Genomics 15 Suppl 4, S7 (2014). 25057121 10.1186/1471-2164-15-S4-S7 PMC4083412

[b45] Chatr-Aryamontri A. *et al.* The BioGRID interaction database: 2015 update. Nucleic Acids Res. 43, D470–D478 (2015). 25428363 10.1093/nar/gku1204 PMC4383984

[b46] Hoffmann R. & Valencia A. A gene network for navigating the literature. Nat. Genet. 36, 664 (2004). 15226743 10.1038/ng0704-664

[b47] Pagel P. *et al.* The MIPS mammalian protein–protein interaction database. Bioinformatics 21, 832–834 (2005). 15531608 10.1093/bioinformatics/bti115

[b48] Orchard S. *et al.* Protein interaction data curation: the International Molecular Exchange (IMEx) consortium. Nat Meth 9, 345–350 (2012). 10.1038/nmeth.1931 PMC3703241 22453911

[b49] Consortium T. U. UniProt: a hub for protein information. Nucl. Acids Res. 43, D204–D212 (2015). 25348405 10.1093/nar/gku989 PMC4384041

[b50] Berman H., Henrick K., Nakamura H. & Markley J. L. The worldwide Protein Data Bank (wwPDB): ensuring a single, uniform archive of PDB data. Nucleic Acids Res. 35, D301–D303 (2007). 17142228 10.1093/nar/gkl971 PMC1669775

[b51] Ashburner M. *et al.* Gene ontology: tool for the unification of biology. The Gene Ontology Consortium. Nat. Genet. 25, 25–29 (2000). 10802651 10.1038/75556 PMC3037419

[b52] Finn R. D. *et al.* The Pfam protein families database: towards a more sustainable future. Nucl. Acids Res. 44, D279–D285 (2016). 26673716 10.1093/nar/gkv1344 PMC4702930

[b53] Acland A. *et al.* Database resources of the National Center for Biotechnology Information. Nucleic Acids Res 42, D7–D17 (2014). 24259429 10.1093/nar/gkt1146 PMC3965057

[b54] Ashkenazy H., Erez E., Martz E., Pupko T. & Ben-Tal N. ConSurf 2010: calculating evolutionary conservation in sequence and structure of proteins and nucleic acids. Nucleic Acids Res. 38, W529–W533 (2010). 20478830 10.1093/nar/gkq399 PMC2896094

[b55] Keskin O., Nussinov R. & Gursoy A. PRISM: protein-protein interaction prediction by structural matching. Methods Mol. Biol. 484, 505–521 (2008). 18592198 10.1007/978-1-59745-398-1_30 PMC2685641

[b56] Chelliah V., Blundell T. & Mizuguchi K. Functional restraints on the patterns of amino acid substitutions: application to sequence-structure homology recognition. Proteins 61, 722–731 (2005). 16193489 10.1002/prot.20617

[b57] Dinkel H. *et al.* The eukaryotic linear motif resource ELM: 10 years and counting. Nucleic Acids Res. 42, D259–D266 (2014). 24214962 10.1093/nar/gkt1047 PMC3964949

[b58] Marco Biasini. PV-WebGL-based protein viewer. doi: 10.5281/zenodo.12620 (2014).

[b59] Wheeler D. A. & Wang L. From human genome to cancer genome: The first decade. Genome Res. 23, 1054–1062 (2013). 23817046 10.1101/gr.157602.113 PMC3698498

[b60] Wang E. *et al.* Predictive genomics: A cancer hallmark network framework for predicting tumor clinical phenotypes using genome sequencing data. Seminars in Cancer Biology 30, 4–12 (2015). 24747696 10.1016/j.semcancer.2014.04.002

